# SELF-REPORTED FALL RISK AND FEAR OF FALLING OF OLDER ADULTS: THE MEDIATING AND MODERATING IMPACT OF HEALTH LITERACY

**DOI:** 10.1093/geroni/igae098.4072

**Published:** 2024-12-31

**Authors:** Dahee Kim, Ladda Thiamwong, Christopher Emrich, Yingru Li, Rui Xie

**Affiliations:** University of Central Florida, Orlando, Florida, United States; University of Central Florida, Orlando, Florida, United States; University of Central Florida, Orlando, Florida, United States; University of Central Florida, Orlando, Florida, United States; University of Central Florida, Orlando, Florida, United States

## Abstract

**Objectives**: Older adults who perceive more fall risks in their daily lives may have a higher fear of falling (FOF). Despite, as a human agency, health literacy of older adults may improve their health and well-being outcomes, but the role of health literacy in fall risk-FOF association has been less highlighted. This study investigated mediating and moderating impacts of their health literacy on the fall risk-FOF association. Methods. This study used data from 176 community-dwelling older adults (MeanAge = 74.49, SDAge = 7.16). Self-reported fall risk was measured using the CDC STEADI fall risk checklist. FOF was assessed using the Short Falls Efficacy Scale International. Health literacy was reported as the level of confidence in filling out a medical form by oneself. This study employed the SPSS PROCESS Macro to test the mediation and moderation models. Results. Older adults’ health literacy moderated their self-reported fall risk-FOF association. The more fall risks older adults perceive, the higher FOF the Low health literacy group showed, whereas the lower FOF the High health literacy group reported. This indicates that health literacy would buffer the impact of fall risk in daily life on FOF in late adulthood. However, we found no indirect impact of self-reported fall risk on FOF via health literacy. Conclusion. These findings highlight the role of health literacy in mitigating the impact of self-reported fall risk on FOF of older adults. This study suggests health literacy as an agency that enhances health and well-being in older adults’ remaining lives.

